# Magnetic Resonance Spectroscopy in Patients with Insomnia: A Repeated Measurement Study

**DOI:** 10.1371/journal.pone.0156771

**Published:** 2016-06-10

**Authors:** Kai Spiegelhalder, Wolfram Regen, Christoph Nissen, Bernd Feige, Chiara Baglioni, Dieter Riemann, Jürgen Hennig, Thomas Lange

**Affiliations:** 1 Department of Clinical Psychology and Psychophysiology, Center for Mental Disorders, University Medical Center Freiburg, Freiburg, Germany; 2 Freiburg Institute of Advanced Studies (FRIAS), University of Freiburg, Freiburg, Germany; 3 Department of Radiology, Medical Physics, University Medical Center Freiburg, Freiburg, Germany; Central Institute of Mental Health, GERMANY

## Abstract

Chronic insomnia is one of the most prevalent central nervous system disorders. It is characterized by increased arousal levels, however, the neurobiological causes and correlates of hyperarousal in insomnia remain to be further determined. In the current study, magnetic resonance spectroscopy was used in the morning and evening in a well-characterized sample of 20 primary insomnia patients (12 females; 8 males; 42.7 ± 13.4 years) and 20 healthy good sleepers (12 females; 8 males; 44.1 ± 10.6 years). The most important inhibitory and excitatory neurotransmitters of the central nervous system, γ-aminobutyric acid (GABA) and glutamate/glutamine (Glx), were assessed in the anterior cingulate cortex (ACC) and dorsolateral prefrontal cortex (DLPFC). The primary hypothesis, a diurnal effect on GABA levels in patients with insomnia, could not be confirmed. Moreover, the current results did not support previous findings of altered GABA levels in individuals with insomnia. Exploratory analyses, however, suggested that GABA levels in the ACC may be positively associated with habitual sleep duration, and, thus, reduced GABA levels may be a trait marker of objective sleep disturbances. Moreover, there was a significant GROUP x MEASUREMENT TIME interaction effect on Glx in the DLPFC with increasing Glx levels across the day in the patients but not in the control group. Therefore, Glx levels may reflect hyperarousal at bedtime in those with insomnia. Future confirmatory studies should include larger sample sizes to investigate brain metabolites in different subgroups of insomnia.

## Introduction

Chronic insomnia, one of the most prevalent mental disorders, is characterized by difficulties in initiating or maintaining sleep and is associated with clinically significant distress or impairment of daytime functioning [1]. The prevalence of the disorder is approximately 6% of the population in western industrialized countries [2]. Chronic insomnia has a negative impact on quality of life [3], and is associated with cognitive impairments [4]. Furthermore, afflicted individuals have a substantially increased risk to develop psychiatric [5,6,7], and cardiovascular disorders [8,9,10,11]. Consequently, chronic insomnia leads to a substantial increase in health care use, work disability and absenteeism, and, thus, to significant direct and indirect costs [12,13,14].

Despite the negative personal, public health and socio-economic impact of chronic insomnia, its neurobiological causes and consequences are poorly understood [15]. Several lines of evidence suggest that γ-aminobutyric acid (GABA), the most important inhibitory neurotransmitter of the central nervous system, is involved in the development and/or maintenance of insomnia. First, the crucial role of GABAergic neurotransmission for the initiation and maintenance of sleep has been clearly demonstrated in animal research [16]. Moreover, in humans, agonists at the benzodiazepine receptor are approved for the treatment of insomnia, and are the most widely used drugs for this condition [17]. While tolerance and dependence are important side effects of these substances, there is little doubt that benzodiazepine receptor agonists improve sleep continuity in insomnia patients undergoing short-term treatment [18,19].

Last, and maybe most importantly, in vivo measures using magnetic resonance spectroscopy (MRS) suggest that cortical GABA levels are altered in patients with insomnia. MRS is a noninvasive method to assess concentrations of various metabolites in the human brain, including GABA, and the composite measure of glutamate and glutamine (Glx), which reflects the activity of the most important excitatory neurotransmitter system. Winkelman et al. [20] used MRS to investigate 16 patients with insomnia and 16 healthy control participants, and reported almost 30% lower GABA levels in the patients group in a large voxel encompassing subcortical, temporal, parietal, and occipital areas (effect size Cohen's d = 0.8). In a follow-up study, the same research group investigated 20 patients with insomnia and 20 healthy controls with improved methodology. In addition to replicating their previous results, lower GABA levels in the patients group were specifically localized in the anterior cingulate cortex (ACC; Cohen's d = 0.8) and occipital cortex (Cohen's d = 1.4), but not in the thalamus [21]. The finding of reduced GABA levels in the central nervous system of patients with insomnia is also in line with the assumption that the disorder is characterized by increased physiological arousal levels [22]. However, there is also conflicting evidence. Morgan et al. [23] reported increased occipital GABA levels in a group of 16 insomnia patients in comparison with 17 healthy good sleepers.

A critical difference between the studies by the Winkelman group [20,21] and the study by Morgan et al. [23] is the timing of the MRS measurements. While Winkelman et al. [20] and Plante et al. [21] acquired MRS data in the morning, Morgan et al. [23] performed the spectroscopic investigation 2–3 hours before bedtime in the evening. Therefore, it has been hypothesized that there may be diurnal effects on GABA levels in insomnia patients [24]. To further clarify this issue, the current study was designed to investigate morning and evening levels of GABA+ (which includes contributions from macromolecules) in a well-characterized sample of 20 patients with insomnia and 20 healthy controls. As preliminary work at the Department of Radiology of the University Medical Center Freiburg showed high test-retest reliability for the assessment of GABA+ levels in the ACC and dorsolateral prefrontal cortex (DLPFC) using a MEGA-PRESS sequence (Mescher-Garwood point-resolved spectroscopy sequence [25]) in a single subject, the current study focused on these specific brain areas. In addition to this main focus of the study, Glx levels were investigated in an exploratory manner as another potential marker of hyperarousal in insomnia.

## Materials and Methods

### Participants

In the current study, 20 patients meeting diagnostic criteria for primary insomnia (PI), according to DSM-IV-TR [26], and 20 healthy control participants were investigated. Inclusion criteria were the insomnia diagnosis in the patients group as well as an age between 18 and 65 years in all participants. Group matching was done for age and sex. Insomnia patients were referred to our clinic by their primary care provider or medical specialist; healthy controls were recruited through local advertisements. Resting-state functional connectivity data of the sample has been published elsewhere [27]. Six PI patients, all non-remitters to cognitive-behavioral therapy for insomnia, and 19 healthy controls have already participated in previous neuroimaging studies of our research group [28,29,30,31]. For screening purposes, a semi-standardized psychiatric and sleep-related interview was conducted in all participants by an experienced psychiatrist to rule out any lifetime history of psychiatric disorder, or (additional) sleep disorder. In addition, all participants underwent a standard physical examination, including electrocardiogram, electroencephalogram (EEG) and routine blood test (blood cell count, liver, renal and thyroid function) to exclude those with serious medical conditions. Furthermore, two nights of polysomnography (PSG) were used in all participants to exclude additional sleep disorders. All participants were free of any psychoactive medication at least 2 weeks prior to enrollment and during the duration of the study, and were required to refrain from alcohol, caffeine and daytime naps during the recording days.

The study was conducted in accordance with the Declaration of Helsinki. The study protocol was approved by the Institutional Review Board of the University Medical Center Freiburg. All participants gave their informed written consent prior to inclusion in the study.

### Procedure

After the screening procedure and two nights of PSG, the MRS investigations were scheduled between 8:00–9:00 AM and 10:00–11:00 PM at the Department of Radiology of the University Medical Center Freiburg. In each participant, morning and evening MRS measurements were performed on the same day. Of note, for logistic reasons—mainly scanner availability and schedules of participants—the MRS measurements were carried out up to several months after PSG recordings. Psychometric assessment was carried out before the evening MRS session.

### Polysomnography

All participants underwent two consecutive nights of standard PSG sleep monitoring in the sleep laboratory. Sleep was recorded for eight hours from ‘lights out’ (10 to 11 PM) until ‘lights on’ (6 to 7 AM) and was scored visually by experienced raters according to standard criteria [32]. All patients were screened for apneas and periodic leg movements by monitoring abdominal and thoracic effort, nasal airflow, oxymetry, and bilateral tibialis anterior EMG. Sleep recordings were evaluated for the following parameters of sleep continuity: total sleep time (TST); sleep onset latency (SOL; time from lights out to the first epoch of stage 2 sleep); wake after sleep onset (WASO) defined as difference between sleep period time (SPT; time from sleep onset to final awakening) and TST; and arousal index. Sleep architecture parameters were amounts of stages 1, 2, slow wave sleep (SWS) and rapid eye movement sleep (REM) as percentage of SPT.

### Psychometric assessment

All study participants were asked to complete the Insomnia Severity Index (ISI) [33], the Pittsburgh Sleep Quality Index (PSQI) [34], the brief version of the Dysfunctional Beliefs and Attitudes about Sleep Scale (DBAS-16) [35], the Glasgow Sleep Effort Scale (GSES) [36], the Pre-Sleep Arousal Scale (PSAS) [37], the Epworth Sleepiness Scale (ESS) [38], the Morningness-Eveningness Questionnaire (MEQ) [39], the Beck Depression Inventory (BDI) [40], and the trait subscale of the State-Trait Anxiety Inventory (STAI) [41].

### MRS data acquisition and preprocessing

**Fig 1:**
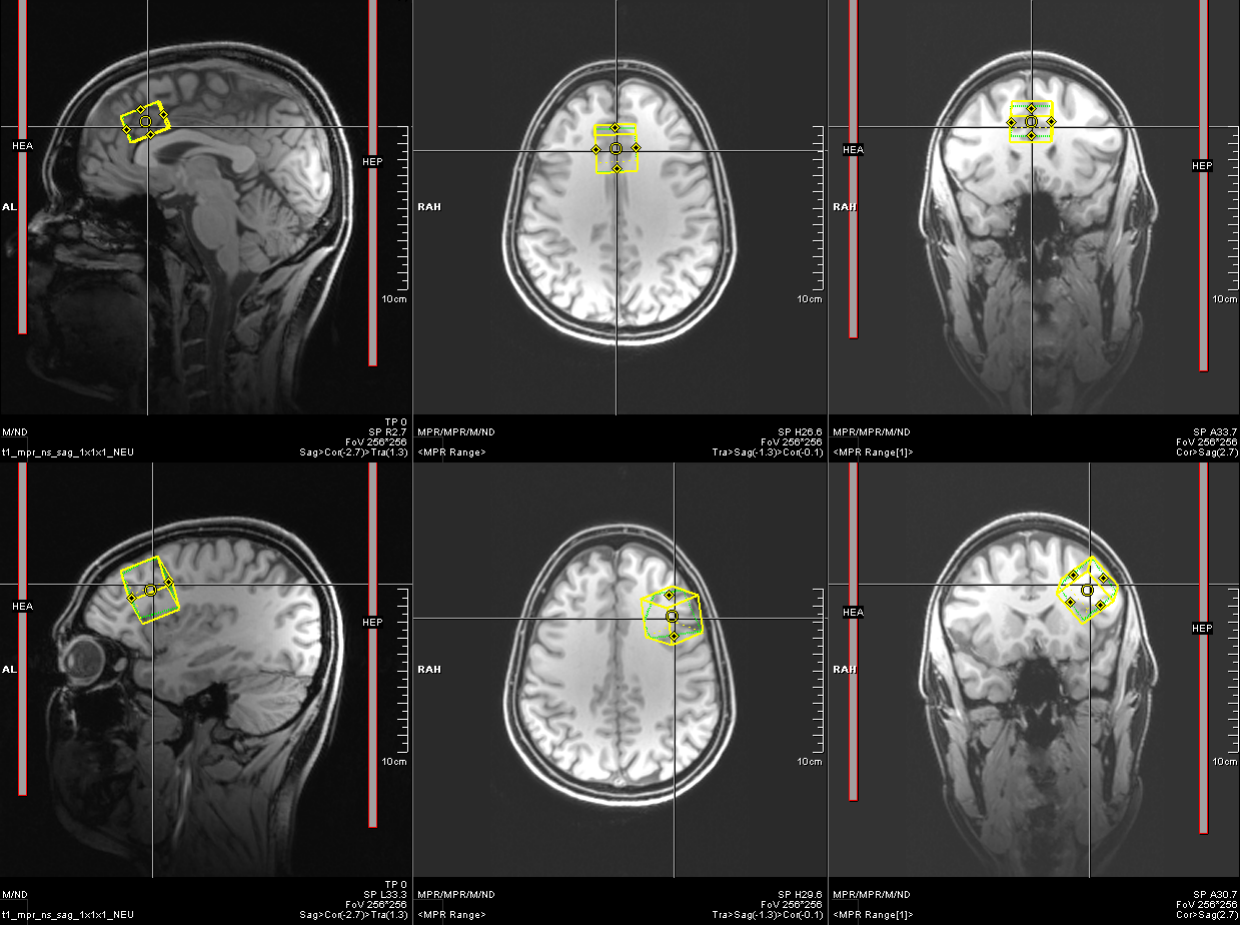
Voxel localization in the ACC (top), and the right DLPFC (bottom) for one participant.

MRS measurements were performed on a 3 Tesla MRI scanner (Magnetom TIM-Trio, Siemens, Erlangen, Germany) using a 32-channel head-coil. First, for accurate MRS voxel positioning, T1 data were acquired using an MPRAGE sequence (TR 2.2 s; TE 2.6 ms; 160 sagittal slices of 256 × 256 voxels, 1.0 × 1.0 × 1.0 mm³; [42]). All scans were inspected for the absence of pathological findings by a neurologist under the supervision of a board-certified neuroradiologist. MRS voxels in the ACC (size: 25 × 25 × 18 mm^3^) and the left DLPFC (size: 25 × 25 × 25 mm^3^) were positioned such that they covered as much gray matter and as little white matter and cerebrospinal fluid as possible (see Fig 1). To avoid strong contamination of the spectra with subcutaneous lipid signal, a safety margin of a few mm was left between the voxel and the skull.

MRS was acquired with the MEGA-PRESS sequence (TE = 68 ms; TR = 1.5 s; 512 averages; measurement time: 13 min per scan). After each MEGA-PRESS scan, a water reference PRESS spectrum was acquired, using the same parameters as for the MEGA-PRESS scan without water suppression (exception: 16 instead of 512 averages). Preparation before the scan included automatic shimming up to second order and was amended by manual shimming to optimize the spectral line width. In total, a single voxel measurement required approximately 20–25 min. Thus, the whole session had a duration of approximately 60 min including MPRAGE image acquisition.

The acquired spectra were fitted and quantified with LCModel [43,44], using numerically simulated basis spectra for the major detectable brain metabolites. The GABA+/NAA concentration ratio was determined from the difference editing spectra while the concentration of all other metabolites was quantified in the unedited spectra of the MEGA-PRESS sequence. Metabolite concentrations were only included in the statistical analysis when their Cramér-Rao lower bounds in the fit were below 20% [45]. This resulted in the exclusion of 3 participants for the analysis of GABA+ in the ACC (2 PI patients, 1 healthy control), 12 participants for the analysis of GABA+ in the DLPFC (5 PI patients, 7 healthy controls), 2 participants for the analysis of Glx in the ACC (2 PI patients), and 2 participants for the analysis of Glx in the DLPFC (2 PI patients). For both GABA+ and Glx, the ratio to creatine (Cr) was calculated for further analysis, which is a standard procedure in clinically oriented MRS research aiming at removing the effects of inter-scan signal intensity variations [46]. Metabolite concentration ratios (GABA+/Cr and Glx/Cr) were corrected for relaxation effects using T1 and T2 relaxation constants of GABA, Glx and Cr obtained from literature [47,48,49,50]. It should be noted that the measured GABA concentration is the sum of several contributions (free GABA, GABA moiety of homocarnosine, and macromolecular resonances of lysine and arginine at 3.0 ppm, which are co-edited by the MEGA-PRESS sequence) and is therefore referred to as GABA+ [51].

### Statistical analysis

Group differences in demographic and polysomnographic data were investigated using two-sample t-tests and chi-squared tests. Pearson correlations between morning and evening measures of GABA+/Cr and Glx/Cr were calculated to assess the short-term stability of these measures. Age- and sex-controlled two-way ANOVAs were used to investigate the impact of GROUP, MEASUREMENT TIME and the GROUP x MEASUREMENT TIME interaction on GABA+/Cr and Glx/Cr levels. Of note, the two-way ANOVA with GABA+/Cr as dependent variable was the primary analysis of this study. The other analyses should be regarded as exploratory. In addition, age- and sex-controlled multiple linear regression analyses were carried out for both groups combined to explore the relationship between GABA+/Cr or Glx/Cr with the following sleep-related variables: polysomnographically determined TST, SOL, SWS %, and REM %, ISI scores, PSQI scores, and MEQ scores. The level of significance was set at p < 0.05 (two-tailed) for all analyses.

## Results

### Sample characteristics

Demographic characteristics and psychometric data of the study sample are presented in Table 1. The groups did not differ significantly in sex distribution, age or body mass index (BMI). The PI patients had significantly higher scores on the ISI, PSQI, DBAS-16, GSES, PSAS, and the trait subscale of the STAI. Furthermore, BDI scores were increased in PI patients, and the group difference was reduced to a statistical trend when excluding the two sleep-related questions from the BDI (PI patients: 4.6 ± 3.6, good sleeper controls: 2.5 ± 3.2, t = -2.00, p = 0.052). No significant group differences were found for MEQ and ESS scores. Two insomnia patients suffered from sleep-onset insomnia, 9 from sleep-maintenance insomnia, and 9 from mixed insomnia. The average duration of primary insomnia was 9.4 ± 10.0 years.

**Table 1:** Description of the study population (means ± standard deviations).

	PI patients	healthy controls	t/χ²	p
Sex [M/F]	8/12	8/12	0.00	1.000
Age [years]	42.7 ± 13.4	44.1 ± 10.6	-0.37	0.716
BMI [kg/m²]	24.5 ± 3.8	23.4 ± 3.3	0.97	0.339
ISI	12.8 ± 3.1	1.6 ± 1.9	13.62	<0.001
PSQI	10.0 ± 3.5	2.6 ± 1.8	8.32	<0.001
DBAS-16	4.5 ± 1.4	2.0 ± 1.2	6.41	<0.001
GSES	5.5 ± 2.6	1.0 ± 1.2	6.88	<0.001
PSAS—cognitive	19.2 ± 6.8	10.6 ± 2.6	5.30	<0.001
PSAS—somatic	11.5 ± 3.2	9.1 ± 1.3	3.04	0.005
ESS	8.0 ± 4.3	5.6 ± 3.3	1.97	0.057
MEQ	54.9 ± 12.9	58.6 ± 7.9	-1.12	0.272
BDI	6.5 ± 3.9	3.1 ± 3.6	2.88	0.006
STAI—trait	38.5 ± 8.6	29.3 ± 4.4	4.16	<0.001

PI: primary insomnia; BMI: body mass index; ISI: Insomnia Severity Scale; PSQI: Pittsburgh Sleep Quality Index; DBAS: Dysfunctional Beliefs and Attitudes about Sleep Scale; GSES: Glasgow Sleep Effort Scale; PSAS: Pre-Sleep Arousal Scale; ESS: Epworth Sleepiness Scale; MEQ: Morningness-Eveningness Questionnaire; BDI: Beck Depression Inventory; STAI: State-Trait Anxiety Inventory.

### Polysomnography

Polysomnographic data are presented in Table 2. In both nights, PI patients had a significantly lower total sleep time and sleep efficiency compared to healthy controls. Additionally, PI patients had a significantly increased SOL and WASO in the first night and a significantly decreased SWS % in the second night.

**Table 2:** Polysomnographic data (means ± standard deviations).

**First night:**	**PI patients**	**healthy controls**	**t**	**p**
Total sleep time [min]	348.2 ± 78.7	397.9 ± 47.0	-2.38	0.023
Sleep efficiency [%]	72.7 ± 16.6	82.8 ± 9.7	-2.30	0.027
Sleep onset latency [min]	30.2 ± 23.9	16.2 ± 13.8	2.23	0.032
WASO [min]	97.0 ± 60.6	61.1 ± 39.0	2.19	0.035
Arousal index / TST [h^-1^]	18.4 ± 7.4	18.0 ± 7.5	0.20	0.846
AHI [h^-1^]	0.3 ± 0.6	0.5 ± 0.7	-0.89	0.379
PLMS arousal index [h^-1^]	0.6 ± 0.9	0.4 ± 0.8	0.60	0.553
Stage 1 [% SPT]	9.2 ± 3.3	10.7 ± 4.9	-1.19	0.242
Stage 2 [% SPT]	49.8 ±9.7	51.4 ± 9.6	-0.53	0.602
SWS [% SPT]	5.1 ± 5.8	7.4 ± 6.1	-1.21	0.233
REM [% SPT]	13.6 ± 6.2	17.0 ± 4.0	-2.01	0.052
**Second night:**	**PI patients**	**healthy controls**	**t**	**p**
Total sleep time [min]	399.7 ± 32.1	422.7 ± 19.0	-2.68	0.011
Sleep efficiency [%]	83.4 ± 6.7	87.8 ± 3.9	-2.42	0.021
Sleep onset latency [min]	17.9 ± 14.8	13.3 ± 7.9	1.20	0.238
WASO [min]	49.1 ± 24.6	43.4 ± 17.2	0.83	0.411
Arousal index / TST [h^-1^]	15.9 ± 8.1	13.8 ± 4.6	0.96	0.343
Stage 1 [% SPT]	8.4 ± 3.9	7.9 ± 4.1	0.38	0.704
Stage 2 [% SPT]	55.1 ± 7.3	53.7 ± 6.3	0.65	0.517
SWS [% SPT]	6.0 ± 6.0	10.2 ± 6.4	-2.10	0.043
REM [% SPT]	19.5 ± 4.9	18.9 ± 4.2	0.43	0.666

PI: primary insomnia; WASO: wake after sleep onset; TST: total sleep time; AHI: apnea hypopnea index; PLMS: periodic leg movements during sleep; SPT: sleep period time; SWS: slow wave sleep; REM: rapid eye movement sleep.

### Magnetic Resonance Spectroscopy

**Fig 2:**
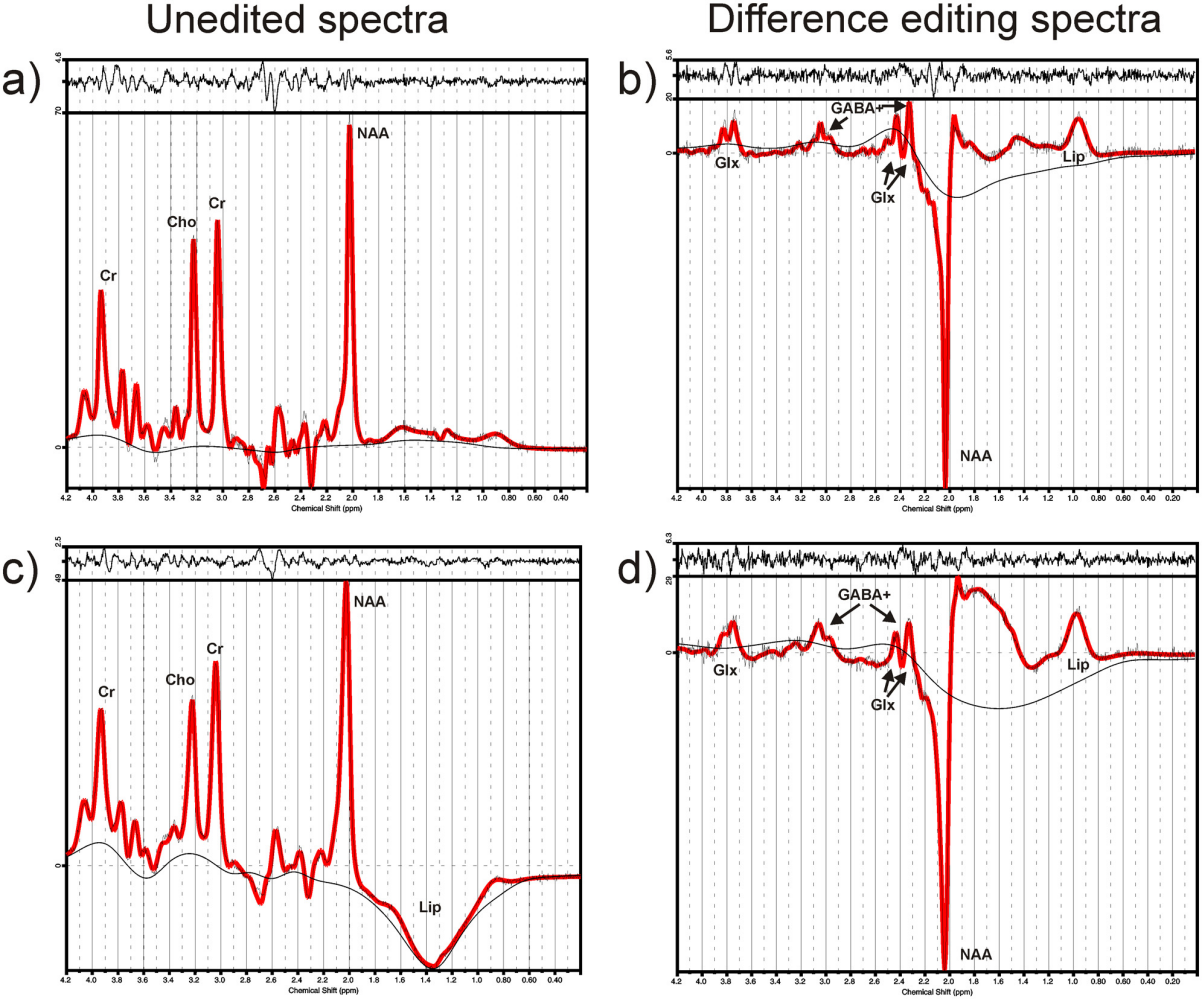
Unedited and difference-edited spectra from the ACC (a, b) and the DLPFC (c, d) of one healthy control participant, as fitted with LCModel: the fitted spectrum (red line), the fitted baseline, and the fit residue (top) are shown.

Fig 2 shows representative unedited as well as difference-edited spectra acquired from one healthy control participant in the ACC and DLPFC. While the unedited spectra are dominated by the large singlet resonances of NAA, Cr and choline, the difference editing spectra show resonances of NAA, GABA+, Glx and lipids. The NAA resonance appears in the difference editing spectra since it is directly affected by the frequency-selective editing pulse irradiated at 1.9 ppm. GABA+, Glx and lipids resonances were edited (GABA+) or co-edited (Glx, lipids) through J-coupling to a resonance affected by this editing pulse. The unedited DLPFC spectra show larger lipid contamination in the spectral region between 0.9 and 2.0 ppm than the ACC spectra since the DLPFC voxel was located close to the skull. Average linewidths of 4.9 Hz and 5.5 Hz were determined by LCModel for the ACC and DLPFC spectra, respectively. The slightly better shim in the ACC measurements is mainly due to the larger DLPFC voxel size. The larger number of DLPFC measurements that had to be excluded for the GABA+ analysis can be explained with the larger linewidths and stronger lipid contamination compared to the ACC measurements.

### GABA+

**Fig 3:**
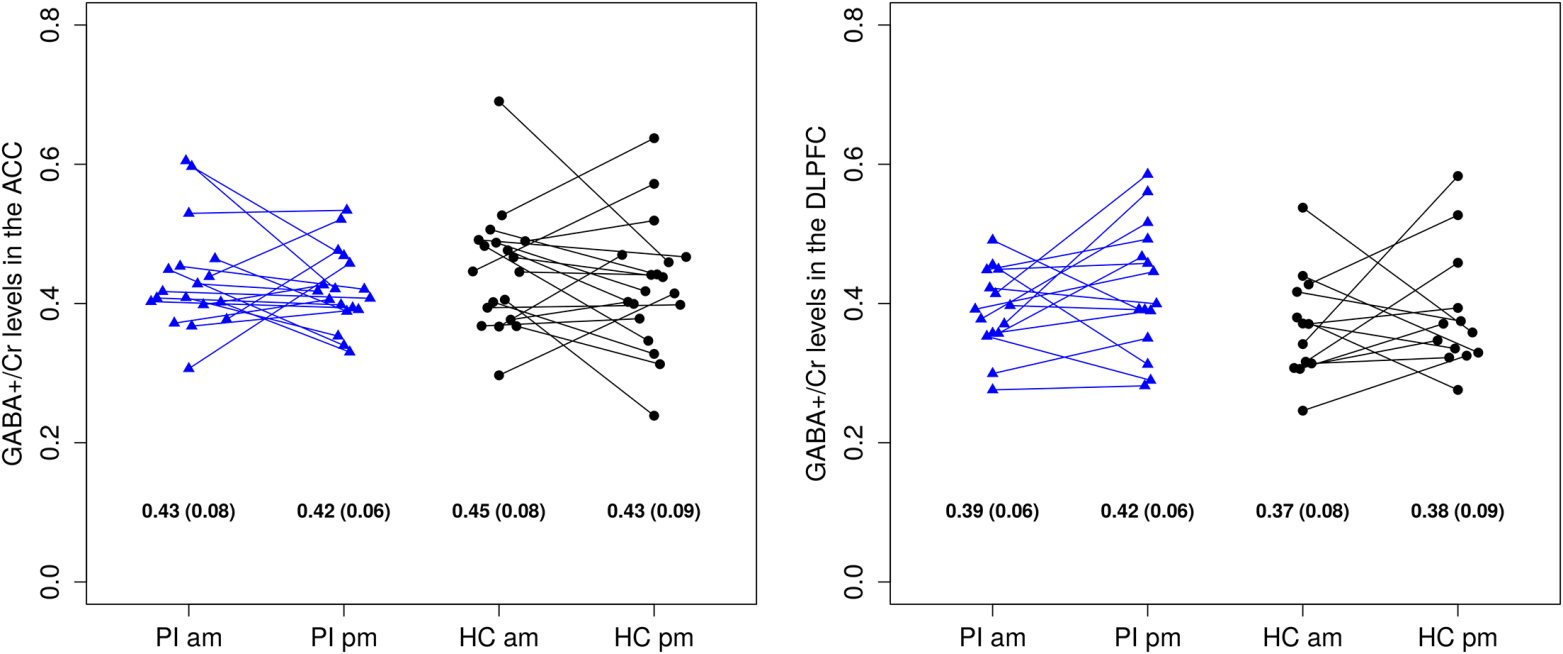
GABA+/Cr concentration ratios in the ACC (left) and DLPFC (right) sorted by group and measurement time. There were no significant main (GROUP; MEASUREMENT TIME) or interaction effects (GROUP x MEASUREMENT TIME). For each group and measurement time, means (standard deviations) are presented at the bottom of the figure. GABA+: free GABA, GABA moiety of homocarnosine, and macromolecular resonances of lysine and arginine at 3.0 ppm; Cr: creatine; DLPFC: dorsolateral prefrontal cortex; PI: primary insomnia; am: ante meridiem; HC: healthy controls; pm: post meridiem.

Fig 3 shows the GABA+/Cr concentration ratios as measured with MRS for the PI patients and healthy controls.

There was a significant positive correlation between morning and evening GABA+/Cr levels in the ACC (r[35] = 0.34; p = 0.041), but not in the DLPFC (r[26] = 0.19; p = 0.335). Age- and sex-controlled ANOVAs did not reveal any significant main (GROUP; MEASUREMENT TIME) or interaction effects (GROUP x MEASUREMENT TIME) on GABA+/Cr levels in the ACC or DLPFC (see Fig 3).

Exploratory age- and sex-controlled linear regression analyses revealed a significant positive association between GABA+/Cr levels in the ACC and TST of the second sleep laboratory night (t[31] = 2.28; p = 0.030). Apart from this finding, there were no significant main effects of the sleep-related variables or interaction effects between the sleep-related variables and MEASUREMENT TIME on GABA+/Cr levels (see Table 3). Importantly, one finding on a p < 0.05 level is close to chance level in this series of 22 exploratory analyses. Age and sex did not have a significant impact on the GABA+/Cr ratios in any of the ANOVAs or linear regression analyses.

**Table 3:** Results from the analysis of the relationship between polysomnographic parameters and GABA+/Cr in the ACC and DLPFC.

	GABA+/Cr in the ACC			GABA+/Cr in the DLPFC		
	β	t	p	β	t	p
TST (1. Night)	6.6 x 10^−5^	0.34	0.738	3.3 x 10^−5^	0.14	0.887
TST (2. Night)	1.1 x 10^−3^	2.28	0.030	2.2 x 10^−4^	0.34	0.738
SOL (1. Night)	1.8 x 10^−4^	0.28	0.782	-4.2 x 10^−4^	-0.56	0.578
SOL (2. Night)	-7.9 x 10^−4^	-0.65	0.521	-1.6 x 10^−4^	-0.13	0.897
SWS (1. Night)	1.2 x 10^−3^	0.37	0.711	-8.6 x 10^−4^	-0.29	0.777
SWS (2. Night)	1.4 x 10^−3^	0.60	0.551	2.5 x 10^−4^	0.09	0.927
REM (1. Night)	5.9 x 10^−4^	0.24	0.816	1.3 x 10^−4^	0.05	0.964
REM (2. Night)	4.7 x 10^−3^	1.51	0.140	-5.0 x 10^−4^	-0.14	0.894
ISI	-1.9 x 10^−3^	-0.86	0.397	3.0 x 10^−4^	0.11	0.914
PSQI	-3.1 x 10^−3^	-1.05	0.301	1.9 x 10^−3^	0.57	0.573
MEQ	9.1 x 10^−4^	0.68	0.499	5.2 x 10^−4^	0.30	0.769

GABA+: free GABA, GABA moiety of homocarnosine, and macromolecular resonances of lysine and arginine at 3.0 ppm; Cr: creatine; ACC: anterior cingulate cortex; DLPFC: dorsolateral prefrontal cortex; β: regression coefficients; TST: total sleep time; SOL: sleep onset latency; SWS: slow wave sleep; REM: rapid eye movement sleep; ISI: Insomnia Severity Index; PSQI: Pittsburgh Sleep Quality Index; MEQ: Morningness-Eveningness Questionnaire.

### Glutamate / glutamine

**Fig 4:**
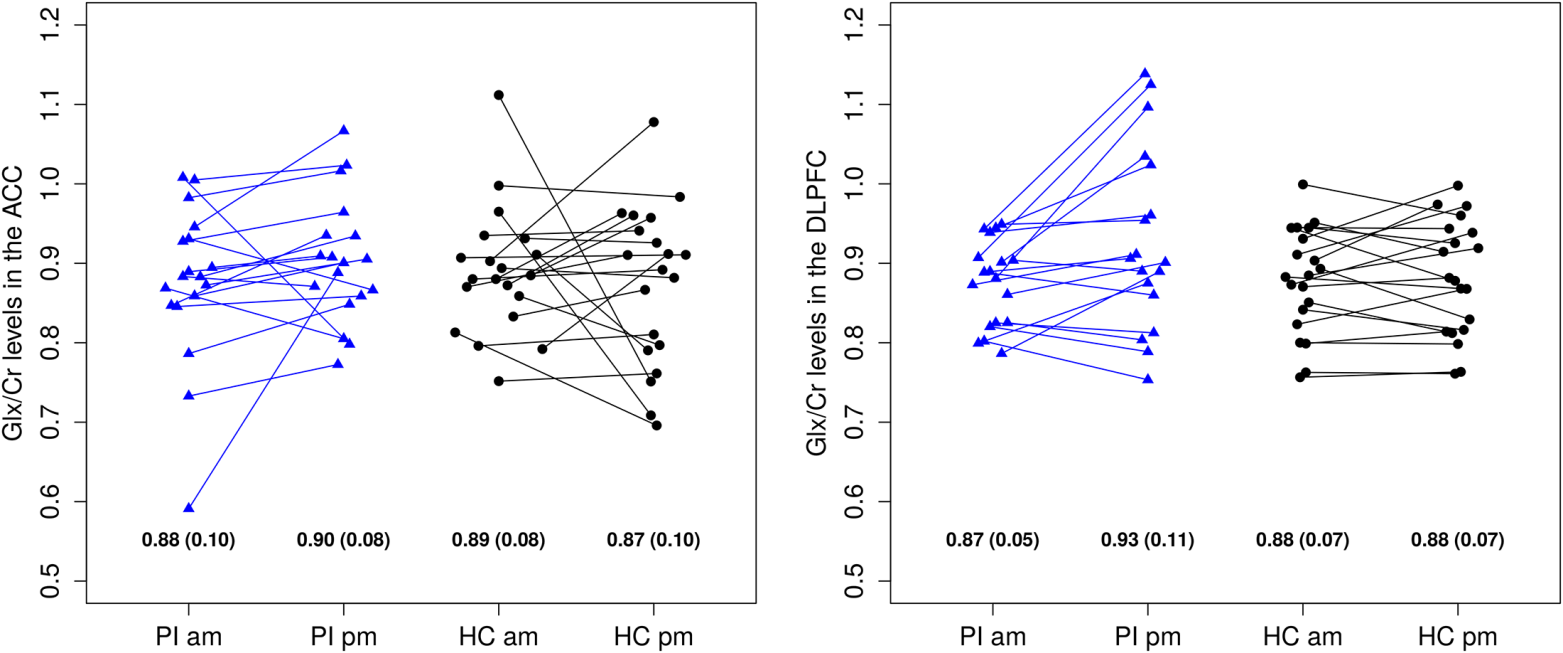
Glx/Cr concentration ratios in the ACC (left) and DLPFC (right) sorted by group and measurement time. There was a significant interaction effect GROUP x MEASUREMENT TIME on Glx/Cr levels in the DLPFC (t[36] = 2.35; p = 0.024). For each group and measurement time, means (standard deviations) are presented at the bottom of the figure. Glx: glutamate and glutamine; Cr: creatine; DLPFC: dorsolateral prefrontal cortex; PI: primary insomnia; am: ante meridiem; HC: healthy controls; pm: post meridiem.

Fig 4 shows the Glx/Cr concentration ratios as measured with MRS for the PI patients and healthy controls. Glx levels were positively correlated across measurement times in the DLPFC (r[36] = 0.68; p < 0.001), but not in the ACC (r[36] = 0.21; p = 0.217). Exploratory age- and sex-controlled ANOVAs did not reveal any significant main effects (GROUP; MEASUREMENT TIME) on Glx/Cr levels in the ACC or DLPFC, however, there was a significant GROUP x MEASUREMENT TIME interaction effect on Glx/Cr in the DLPFC (t[36] = 2.35; p = 0.024). Fig 4 shows that the source of the interaction effect was an increasing Glx/Cr level in the patients group across the day which was not found in the control group.

Exploratory age- and sex-controlled linear regression analyses revealed significant interaction effects for ISI x MEASUREMENT TIME (t[36] = 3.11; p = 0.004) and PSQI x MEASUREMENT TIME (t[36] = 2.46; p = 0.019) on Glx/Cr levels in the DLPFC, but no other significant main or interaction effects. The significant interaction effects were equivalent to the GROUP x MEASUREMENT TIME interaction effect, i.e. increasing Glx/Cr levels across the day were only evident in those with high ISI/PSQI scores. Age had a significant negative effect on the Glx/Cr ratios in both the ACC and DLPFC (p < 0.01 in all ANOVAs and linear regression analyses). Sex did not have a significant impact on the Glx/Cr ratios in any of the analyses.

## Discussion

This is the first study in patients with chronic insomnia investigating brain metabolites with MRS both in the morning and evening. Our primary hypothesis, a diurnal effect on GABA+ levels in patients with insomnia, could not be confirmed. Moreover, the results do not support that altered GABA+ levels are a key characteristic of insomnia. Exploratory analyses, however, suggest that GABA+ levels may be positively associated with habitual sleep duration and Glx levels may reflect increasing arousal levels across the day in those with insomnia.

The hypothesis of diurnal effects on GABA+ levels in patients with insomnia appeared to be a plausible explanation for the inconsistent findings of previous studies [20,21,23]. However, this hypothesis has never been directly investigated before, and can be quite confidently rejected in light of the current results. In both groups, GABA+ levels were relatively stable across the day which supports previous data on the high reproducibility of this measure in healthy individuals [52,53,54]. As a direct consequence of the presented data, future studies should investigate alternative explanations for the discrepancies in the literature including a more detailed assessment of the effects of sample composition and voxel positioning on GABA levels in insomnia [24].

Unfortunately, with respect to the investigation of group differences in GABA+ levels, the current study adds to the inconsistent picture emerging from neuroimaging studies in insomnia [15,55]. Unlike previous studies [20,21,23], there were no significant differences between patients with insomnia and healthy controls. Similar to the arguments presented above, discrepancies in sample composition and in the specific methods for MRS data collection and metabolite quantification across studies may explain these results. While it is obvious that these factors need to be further investigated, it should also be noted that the current study was powered to detect medium effect sizes for the interaction effects GROUP x MEASUREMENT TIME in the repeated measurement analyses. This, however, results in a rather low power for the between-subject analyses, which, unfortunately, has been identified as a ubiquitous limitation in neuroscience [56]. In addition, newly discovered effects often provide inflated effect sizes compared to true effect sizes [57]. Thus, future studies in this field should include larger sample sizes to estimate the association between insomnia and GABA levels with reasonable accuracy. In addition, larger sample sizes may allow for the investigation of sub-groups of insomnia. Of note, given the current sample size as well as observed standard deviations and correlations between repeated measures, effect sizes of f = 0.39 (ACC) and f = 0.42 (DLPFC) could have been detected at an alpha level of p < 0.05 with a power of 80% for the between-subjects factor GROUP in the ANOVA models for GABA+/Cr. This corresponds to absolute between-group differences of 0.069 for both the ACC and DLPFC. For the within-between interaction (GROUP x MEASUREMENT TIME), effect sizes of f = 0.27 (ACC) and f = 0.35 (DLPFC) could have been detected at an alpha level of p < 0.05 with a power of 80%.

Exploratory data analysis revealed a significant positive association between GABA+ levels in the ACC and the total sleep time of the second sleep laboratory night, which is in line with the inhibitory capacity of the GABA system. If this finding turns out to be true, GABA is rather a measure of objective than subjective sleep disturbance giving further support to models postulating that objective sleep disturbances are more closely connected to neurobiological findings than subjective sleep disturbances [58]. Further exploratory analyses suggest that Glx, the composite measure of glutamate and glutamine, and thus a marker of the most important excitatory neurotransmitter system, is an indicator of increased arousal levels at bedtime in patients with chronic insomnia. This is in line with the vast literature on hyperarousal in insomnia [22], and the observation that some arousal markers are specifically increased at bedtime or during sleep, e.g. blood pressure [59], or heart rate [60]. The finding of increased Glx levels in the evening in patients with insomnia complements with two previous MRS studies. Meyerhoff et al. [61] found that ISI scores were positively correlated with evening Glx levels in a sample of patients with posttraumatic stress disorder, and Plante et al. [21] did not report significant group differences between patients with insomnia and healthy controls in an MRS study that included measurements in the morning. However, while the significant findings concerning Glx and the association between GABA+ and TST have some plausibility, both need replication before drawing firm conclusions as we did not correct the exploratory analyses for multiple testing.

From a methodological point of view, the established MEGA-PRESS method was used for GABA detection. Relaxation correction was performed to ensure comparability of our data with other data obtained with different methods at different field strengths. While MEGA-PRESS is the most widely used method for GABA MRS, other techniques such as short echo time single voxel MRS without J-editing [62] and 2D J-resolved spectroscopic imaging [20] have also been applied. The average GABA+/Cr levels measured in this study were comparably high which may be attributed to macromolecule contamination [51]. This contamination can be avoided by using editing pulses with smaller bandwidths, which are, however, not compatible with the standard MEGA-PRESS sequence. Near et al. used a dedicated MEGA-SPECIAL sequence for GABA editing with and without macromolecule suppression and showed that macromolecular signal accounts for 46% of the GABA concentration measured with MEGA-PRESS [63]. Furthermore, to date, most MEGA-PRESS studies have been performed without T2 relaxation correction. This is mainly due to the fact that the T2 constant of GABA has only recently been measured in vivo. Using T2_Cr_ = 154 ms and T2_GABA_ = 88 ms [48] for relaxation correction of MEGA-PRESS data acquired with an echo time of 68 ms further increases GABA+/Cr levels by 40%. Data exclusion was performed based on the CRLBs determined in the LCModel fit. It should be noted that the CRLBs are inversely proportional to the signal-to-noise ratio. This can results in an exclusion bias for low concentrations and can therefore reduce observed differences between groups. However, the number of excluded datasets was about the same for PI patients and healthy controls.

Several limitations of the current investigation have to be acknowledged. First, as the study had a cross-sectional design, conclusions about causality can not be drawn. Therefore, the direction of the putative associations from the exploratory analyses remains unclear. Second, in comparison to previously published studies on GABA levels in insomnia [20,21,23] our sample had a comparably low insomnia severity which may have rendered group differences non-significant. Third, the diurnal stability of GABA+/Cr levels in the current study was somewhat lower than previously reported [54] which may indicate higher error variance in the current data. Fourth, the relative timing of MRS measurements and PSG recordings was not standardized with MRS measurements being carried out up to several months after the PSG recordings. Fifth, we did not control for GABA changes across the menstrual cycle in women [64]. However, Bartlett tests of homogeneity of variances did not suggest increased variances in GABA+/Cr levels in women compared to men (please refer to the original data deposited to the University Library of the University of Freiburg database). Sixth, although none of our participants suffered from a substance dependence disorder, the results of the current study may have been subtly affected by substance discontinuation as all participants were required to refrain from alcohol and caffeine during the recording days. Last, it should be noted that a large number of participants (12) had to be excluded from the analyses of GABA+ in the DLPFC because the Cramér-Rao lower bounds were at least 20%. Thus, statistical power of these analyses is considerably reduced compared to the other analyses.

In summary, our study suggests that diurnal effects on GABA+ levels in patients with insomnia do not explain the inconsistent findings in the literature. In addition, exploratory findings of a positive association between GABA+ and TST of the second sleep laboratory night as well as the finding of increased Glx levels in patients in insomnia in the evening warrant further investigation.
